# Neural Network-Based Shape Analysis and Control of Continuum Objects

**DOI:** 10.3390/biomimetics9120772

**Published:** 2024-12-18

**Authors:** Yuqiao Dai, Shilin Zhang, Wei Cheng, Peng Li

**Affiliations:** 1 School of Mechanical Engineering and Automation, Harbin Institute of Technology Shenzhen, Shenzhen 518055, China; sy2303802@buaa.edu.cn (Y.D.); 23s153088@stu.hit.edu.cn (S.Z.); 2 Devol Advanced Automation, Inc., Jiaan Science Park, Liuxian 1st Road, Bao an District, Shenzhen 518101, China

**Keywords:** continuum object, shape analysis, shape control, neural networks

## Abstract

Soft robots are gaining increasing attention in current robotics research due to their continuum structure. However, accurately recognizing and reproducing the shape of such continuum robots remains a challenge. In this paper, we propose a novel approach that combines contour extraction with camera reconstruction to obtain shape features. Neural networks are employed to model the relationship between motor inputs and the resulting shape output. A simulation environment is established to verify the shape estimation and shape control of the flexible continuum. The outcomes demonstrate that this approach effectively predicts and reproduces the shape of flexible continuum robots, providing a promising solution for continuum shape control.

## 1. Introduction

Small tubular surgical instruments are widely used in minimally invasive surgery. The surgeon manipulates these instruments using images provided by the endoscope to perform the procedure. Different from conventional rigid robots, flexible instruments introduce redundant degrees of freedom, making it more difficult to calculate their position. This complicates real-time shape perception and accurate three-dimensional (3D) modeling. Precise shape perception of the robot is critical in surgery, as it provides the surgeon with information that cannot be obtained visually.

Various flexible continuum robots have been developed to perform complex tasks in robot-assisted minimally invasive procedures [1]. According to [1], these continuum robots can be categorized into several types, including cable-driven robots [2,3], concentric tubular robots [4,5], catheters [6,7], soft robots [8,9], flexible needles [10,11], fluid-operated robots [12,13], and shape memory alloy manipulators [14,15]. Creating a kinematic model of these flexible structures in 3D space enables real-time shape perception of these instruments. Arata et al. presented a four-degree-of-freedom flexible robotic arm in [16]. This robotic arm demonstrates high accuracy and repeatability, distinguishing it from previous designs. The authors successfully modeled its kinematics and verified its performance through elastodynamic methods. Subsequently, in [17], Camarillo and Loewke introduced a vision-based method for real-time 3D shape perception of flexible robots. Their method uses sampling point measurements to improve the precision of 3D shape estimation, contrasting with sensor-based methods commonly used in traditional rigid robotic arms. The correctness of their results was verified through experiments under open-loop control. Loutfi et al. evaluated the performance of four different learning models for the forward kinematics of multi-segment continuous manipulators within the framework of forward kinematics, drawing conclusions for various scenarios in [18].

Recently, a neural network-based method has been presented to solve the forward and inverse kinematics issue, accounting for non-negligible mass and elasticity in [19]. Simulation experiments were performed in a redundant constraint plane and a minimum constraint space to compare their method with conventional approaches. In [20], Godage et al. developed a kinematic model for a multi-segment continuous arm based on mode shape functions (MSF). They successfully applied modal approximation techniques to create a new kinematic model for a generalized variable-expansion, multi-segment continuum robot arm. It illustrates the model’s capability to simulate both spatial bending and straight-arm motions, introducing inverse kinematics for multi-segment continuum systems. An approach for reconstructing the 3D shape of minimally invasive surgical devices has been introduced to enhance the navigation of interventional tools in [21]. Their method integrates physics-based simulation with a nonlinear Bayesian filter, where the physics-based model predicts the shape, and the filter uses external image features to refine the navigation model.

In [22], an actor-critic design has been presented to address tracking control challenges in jointed robots with uncertainties. Neural networks were utilized to handle uncertainty, allowing for the approximation of motion control in these elastic systems. In [23], an approach for tracking and shape reconstruction of wire-driven flexible robots has been designed. Their approach utilizes a third-order Bessel curve-based algorithm, which reconstructs the robot’s shape using position and orientation data acquired from an electromagnetic sensor. The method demonstrated excellent tracking performance and high accuracy in shape reconstruction. Zou et al. proposed an algorithm for 3D shape reconstruction of soft robotic arms based on neural networks in [24]. Their method employs a deep neural network (DNN) to correct the continuum robot’s kinematics through precise visual estimation. The performance of the trained DNN was evaluated on both test sets and real-time bending deformation experiments. The results demonstrated that the DNN can accurately and stably learn the 3D shape of the robotic arm. Recently, an approach utilizing soft e-textile resistive sensors was proposed in [25], offering seamless integration with the robot’s structure. A deep Convolutional Neural Network (CNN) is employed to decode sensor signals, enabling precise shape estimation and control. Unlike the notable results achieved with e-textile sensors in [25], this work focuses on control based on visual feedback.

In this paper, we adopt a neural network-based kinematic modeling approach for shape estimation. First, the flexible continuum’s centerline is extracted from the camera using a contour extraction algorithm. The 3D coordinates of this centerline are then reconstructed using a binocular camera system. These coordinates provide a dataset that describes essential shape information, which is used to train two neural networks. Finally, these networks are trained to accurately estimate and control the shape of the robotic arm.

The remainder of this paper is organized as follows. Section 2 introduces the edge centerline extraction (ECE) algorithm. In Section 3, neural networks are applied to model the input-output relationship of the instrument. Simulation and experimental results are presented in Section 4. Finally, Section 5 offers conclusions based on the findings.

## 2. ECE Algorithm

### 2.1. Pre-Process Image

First, color thresholding segmentation is performed in the HSV color space, employing color information to eliminate irrelevant objects quickly. Next, a joint bilateral filter is applied to eliminate residual noise, ensuring the retention of critical contour edge details. Following this, the Canny operator is employed to extract image contours, producing a binarized contour map and identifying all contour point coordinates.

### 2.2. Self-Organizing Map Algorithm for Contour Centerline Extraction

Self-organizing map (SOM) is a neural network model based on unsupervised learning. It is particularly useful for generating a low-dimensional, discrete map by learning from input data, making it a form of dimensionality reduction. Unlike traditional neural networks that rely on backpropagation to minimize loss functions, SOMs employ competitive learning, where neurons compete to adjust their weights. This approach helps maintain the topology of the input space by using a nearest-neighbor function, as illustrated in Algorithm 1.
**Algorithm 1** ECE Algorithm**Function:**   Centerline Extraction (Image, Contour Point)Initialize SOM**For** k=0,1,2,…,T            Select a contour point D(t) randomly         Identify the distance in the SOM D(t) nearest neuron *v*. Denote it by *u*      **For** all neurons in the SOM                Update weights using (1)      **End****End**Extract neuron weights *W* as centroidsOutput *W*.

The SOM architecture consists of two layers: the input layer and the output layer. The number of neurons in the input layer is determined by the dimensionality of the input vector. In this case, because the input features consist of pixel coordinates (x,y), the input layer is represented in two dimensions. The output layer, or competitive layer, can be either one-dimensional or two-dimensional. A one-dimensional structure forms a line, while a two-dimensional structure creates a grid. For this task, where the goal is to extract a centerline contour (a line), we adopt a one-dimensional structure with 10 neurons to fit the centerline [26,27]:

(1) $$W_{u}\left(k+1\right)=W_{u}\left(k\right)+\theta \left(u,v,k\right)\alpha \left(k\right)\left(D\left(t\right)-W_{u}\left(k\right)\right)$$

where *k* is the step index, *v* is the index of the node. The weights of the node at position *u* are represented as $W_{u}\left(k+1\right)$ at the next training step and $W_{u}\left(k\right)$ at the current training step. θ(u,v,k) is the neighborhood function, with a bubble function applied in this case. The learning rate α(k) is given by:

(2) $$\alpha \left(k\right)=\frac{lr}{1+\frac{k}{T}}.$$

Here, lr is the learning rate and *T* is half of the total training time. D(t) is a target input data vector.

### 2.3. Curve Fitting

After training the network, keypoints are extracted to aid in reconstructing the centerline shape. These keypoints are used to generate a smooth curve through interpolation or fitting techniques. In control theory, the curves generated by PID controllers tend to be smoother. By adjusting the damping ratios, we can achieve different curve effects, which we applied to the spline curves. A two-dimensional PID controller is employed, where the coordinates of the keypoints serve as the control objects, and the positions of the pixel points act as the inputs. A time axis is introduced to describe the states of the points over time. The spline curve is then constructed by projecting the points across all time steps onto the image.

**Figure 1:**
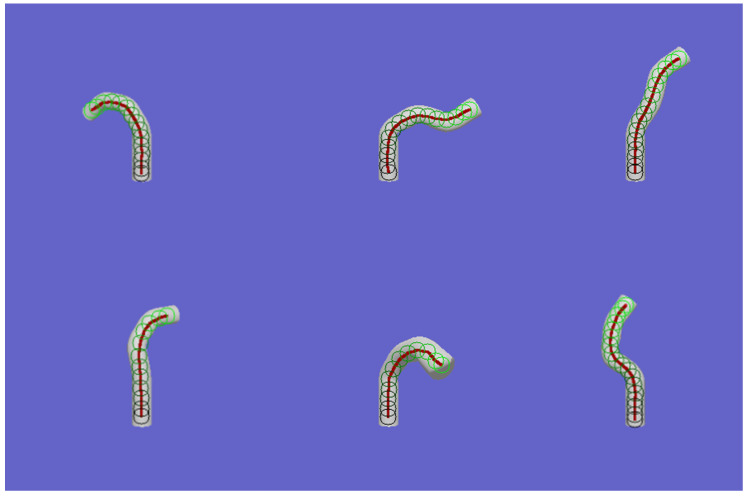
Centerline extraction and spline curve fitting results. The green circles are the extracted center points of the robot.

Starting from the initial state defined by the first keypoint, a reference input is generated by interpolating between the first and second keypoints using time-based weighting. As the control point moves closer to the second keypoint or reaches the threshold time, interpolation shifts to the second and third keypoints to produce the next reference input. This process repeats iteratively until the control point approaches the final keypoint or the threshold time limit is reached. By mapping the control points at each time step onto the image, the resulting spline curve is obtained. The results of centerline extraction and spline curve fitting for six different bending states are presented in Figure 1.

### 2.4. 3D Reconstruction Using a Binocular Camera

Since depth information is not directly available, we utilize binocular camera matching to determine the 3D coordinates. This is achieved by applying the ray intersection algorithm, using the intrinsic camera parameters and the corresponding centroids identified in the two images. The centroid’s world coordinates are calculated through the following transformation:

(3) $$T_{world}=T_{cam}+Q_{cam}\times P_{cam}.$$

Here, $T_{world}$ represents the centroid’s coordinates in the world coordinate system (WCS). $T_{cam}$ denotes the position of the camera focal point’s position in WCS, $Q_{cam}$ is the matrix that represents the camera’s orientation in WCS, and $P_{cam}$ represents the coordinates of the centroid in the camera coordinate system. Mapping the irradiance of robots in space onto the corresponding pixels on the screen, using the perspective transformation matrix, we can project the vertex positions of each triangle in the model onto their corresponding positions on the camera screen, simulating the process of a binocular camera capturing an image.

## 3. Neural Networks

Using contour centerline extraction and 3D reconstruction with binocular cameras, we obtain spatial data on the instrument’s tip shape under various motor inputs. To achieve a functional mapping between the complete set of motor inputs and the corresponding instrument shapes, we develop a model. Given the intricate nature of this input-output relationship, influenced by numerous factors, traditional modeling approaches prove insufficient. Neural networks, known for their strong generalization capabilities and ability to fit complex functions, provide a more effective solution [28,29,30]. In this paper, we train a neural network on a data subset to effectively model the surgical instrument’s input-output dynamics.

### 3.1. The Selection of Neural Networks

We utilize two neural network models for different tasks: one to interpret the instrument’s shape based on the current motor input and another to regulate the shape by modifying the motor input to achieve a desired shape.

The first network, referred to as the encoder, takes the motor input as its input and outputs a mathematical representation of the instrument’s shape. This network consists of 6 layers with 32 neurons in each hidden layer. The hidden layers use the ELU activation function, and linear activation function is applied to the output layer. Key hyperparameters include a seed value of 1234, a batch size of 4, a learning rate of 0.001, and a total of 500,000 training iterations. The second network, referred to as the decoder, takes as input a mathematical representation of the instrument’s shape and outputs the motor input. This network consists of six layers, with a hidden layer size of 96 neurons. The ELU activation function is used in the hidden layers, and linear activation function is applied to the output layer. The model is trained using a seed value of 1234, a batch size of 4, a learning rate of 0.001, and for 500,000 iterations.

### 3.2. Process Dataset

An essential factor in training a neural network is the quality of the dataset provided. A well-prepared dataset is important for reducing data coupling, preserving key features, and optimizing the network’s training process.

*Data Preprocessing.* Once the shape recognition algorithm identifies the centerline of the instrument, appropriate mathematical transformations can be applied, followed by feature extraction from the raw data. To decouple the data as much as possible, algorithms should be designed to reduce its dimensionality, enhancing the overall efficiency of the data. The relative positions between joints are inherently coupled, meaning that a shift in a parent joint affects its child. However, the relative postures between joints remain independent of one another. To capture this information, we use quaternions to extract and store relative posture data for each joint. We first connect the origin of the coordinate system to the root node to establish the initial vector, and then iteratively connect the center points from the root node to the end node, forming the remaining N−1 vectors. Using a forward kinematics algorithm, we can derive the relationship between the equipment’s posture and its relative position and posture, leading to the following equation:

(4) $$T_{child}-T_{parent}=Q_{parent}P_{offset}$$

(5) $$R_{child}=Q_{parent}^{-1}Q_{child}$$

where $T_{child}$ is the world coordinate of the child joint, $T_{parent}$ is the world coordinate of the parent joint, $Q_{parent}$ is the absolute pose of the parent joint, $Q_{child}$ is the absolute pose of the child joint, $P_{offset}$ is the difference between the initial world coordinates of the child joint and the parent joint, and $R_{child}$ is the relative pose of the child joint and the parent joint.

Physically, this relationship represents the rotation of the child joint around the parent joint, encompassing three degrees of freedom. This can be further broken down into two components: a two-degree-of-freedom rotation, which transitions the current direction vector to the target direction vector (referred to as rotational) [31], and a one-degree-of-freedom spin around the final direction vector (referred to as orientational). While rotation influences both the position and orientation of the child joint, orientation affects only the joint’s posture.

**Figure 2:**
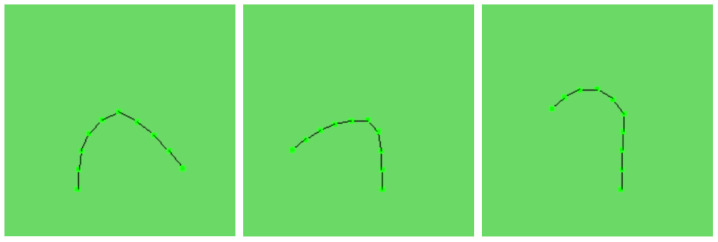
Joint and its associated posture in three different bending states.

After obtaining the center point coordinates, we acquire the position data for all joints. The joint and its associated posture in three different bending states are shown in Figure 2. However, we still lack the pose information necessary to determine the relative orientations of these joints accurately. Since each joint’s movement is constrained, the three rotational degrees of freedom are effectively reduced to two, focusing solely on rotational components. As shown in (4), there is a defined mathematical relationship between the current coordinate difference (i.e., vector) between parent and child joints, the initial coordinate difference, and the absolute pose of the parent joint. Given that only rotational components are involved, the pose can be treated as a rotational transformation. The rotation axis is derived from the cross product of the two vectors, while the dot product yields the rotation angle. We then use the axis-angle relationship to convert this information into a quaternion pose representation. From (5), we can derive the relative orientations of each joint from their absolute orientations, which are represented and computed using quaternion algorithms.

*Data normalization.* Prior to inputting data into the network, it should be normalized by adjusting its mean to 0 and standard deviation to 1. This is done by calculating the overall mean and standard deviation of the dataset, then subtracting the mean from each data point and dividing by the standard deviation.

*Data regularization.* To mitigate overfitting, we introduce noise into the data, with its range determined by the standard deviation of the input data. While this added noise enhances the network’s generalization capability, excessive noise can hinder convergence.

Once the network is trained, the output data are scaled back to its original form by multiplying by the standard deviation and adding the mean.

## 4. Simulation Environment Construction and Experiments

Before conducting physical tests, we verify the algorithm’s feasibility in a simulation environment. This approach, implemented on a computer, minimizes reliance on physical setups and streamlines the testing process. The simulation environment is a simplified representation of the physical world, reducing noise and creating near-ideal conditions. Once the algorithm performs successfully in this virtual setting, it can then be considered for implementation in the physical environment.

### 4.1. Skeletal Animation Environment

In computer graphics, geometric models are typically depicted as surfaces composed of multiple triangles. These triangles store essential information, such as vertex coordinates, normals, and other attributes that define the geometry’s surface characteristics.

The model’s motion is defined by the movement of its vertices, which are bound to a rigid skeleton structure. The root of the skeleton has properties that include spatial position and orientation, while the rest of the skeleton is defined by the relative position and rotation of each segment compared to its parent segment. The relative positions are described by three-dimensional vectors, while orientations and rotations are expressed using quaternions. The motion of the skeleton is governed by forward kinematics, allowing for the iterative calculation of the spatial position and orientation of each skeletal segment. Vertices are connected to bones and move according to the bones’ transformations. If a vertex is influenced by multiple bones, its motion is determined through weighted interpolation of the transformations from each bone.

To begin, a cylinder model is constructed, with multiple sequentially bound bones. Each node on the cylinder is assigned a weight according to their influence by the bones, and their behavior is tested across various angles. For smooth deformation of the flexible structure, weights should be assigned to each vertex appropriately. However, manual weight adjustment becomes impractical due to the large number of vertices. Therefore, we develop an automated algorithm specifically for weight assignment in the flexible body model.

1. Compute the distances from each vertex to all skeletal root nodes and arrange them in ascending order based on proximity.
2. For each vertex, identify the four closest skeletal root nodes and identify their corresponding parent joints. Compute the projection of the vector from the parent joint to the vertex onto the vector from the parent joint to its child. Then, find the ratio of this projection to the relative distance between the parent and child nodes.
3. Derive the influence weight of each vertex based on the calculated vector ratio with respect to the parent joint.
4. Scale the four weights simultaneously to ensure that their total equals 1.

(6) $$f\left(x\right)=\left\{\begin{array}{c} e^{-\frac{{\left(x-0.5\right)}^{2}}{1.5}}\left(x>0.5\right)\\ e^{-\frac{{\left(x-0.5\right)}^{2}}{0.25}}\left(x<0.5\right). \end{array}\right.$$

### 4.2. Image Acquisition

**Figure 3:**
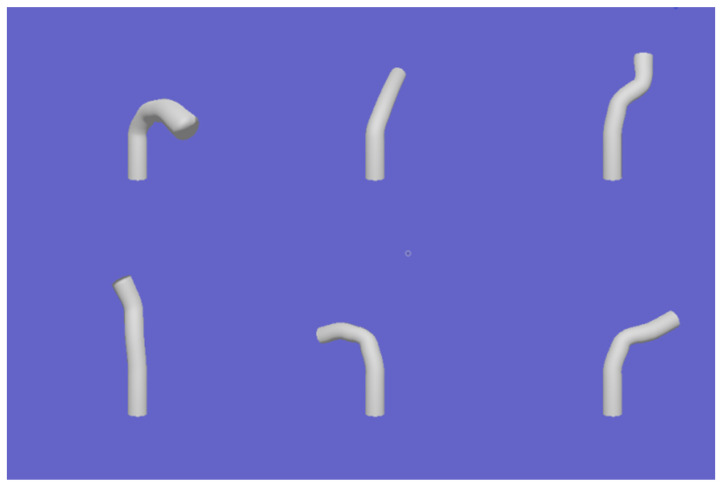
Rendered image of the instrument model.

The model of the instrument is loaded into the rendering environment, where camera settings, lighting, and other parameters are configured before initiating the rendering process. The obtained image is shown in Figure 3.

We simulate motor inputs using a controller’s two joysticks, with each joystick’s X and Y axes representing the inputs for separate motors. This setup enables control of four motors, simulating the deformation of the flexible continuum. The inputs from the controller influence the posture of multiple joints through pre-defined functional relationships, accurately modeling the continuum’s behavior.

Once extraction is complete, a binocular 3D reconstruction algorithm is applied to the center points, yielding the 3D spatial coordinates. After generating substantial raw data using the shape recognition algorithm within the renderer, the datasets are processed according to the method described in Section 3.2 and stored as binary files. These datasets are subsequently used to train both the encoder and decoder networks, following the network parameters outlined in Section 3.1.

### 4.3. Network Training Variance

**Figure 4:**
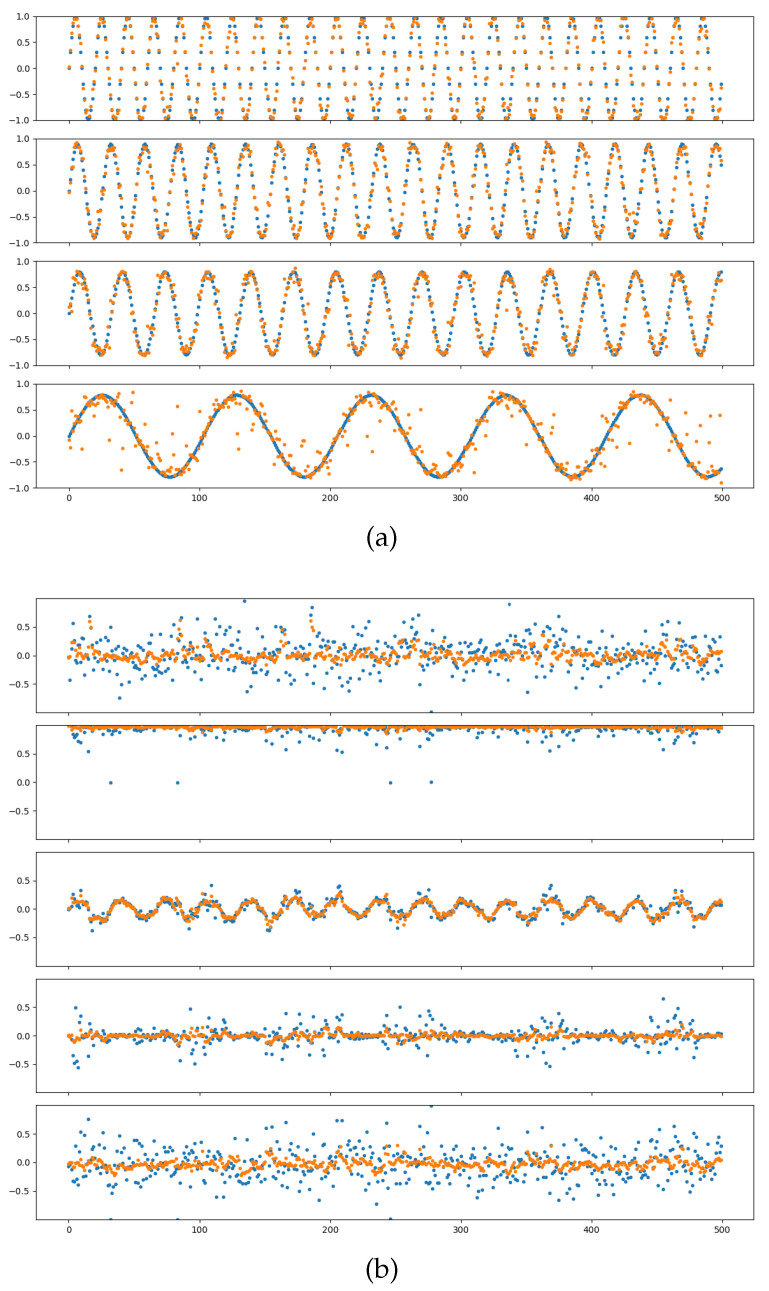
Example of Neural Network Fitting Results: (**a**) Motor Input Fitting: The horizontal axis represents sampling time, while the vertical axis represents input values. The four motor inputs are shown from top to bottom, with yellow indicating actual values and blue indicating fitted values. (**b**) Joint Pose Fitting (Quaternion): The horizontal axis denotes sampling time, and the vertical axis indicates component size. From top to bottom, the components of the quaternion (x, y, z, w) are displayed, with yellow representing actual values and blue representing fitted values.

We performed multiple rounds of network training on the dataset, adjusting the level of noise introduced in each round to develop a stable encoder-decoder model. The results of the training process are presented in Figure 4, where the blue line represents the actual values and the yellow line indicates the fitted values. The figure demonstrates that the neural network effectively captures and fits the complex structures within the data.

Before verifying the model in the simulation environment, we evaluate its performance by calculating the mean square error (MSE) using the verification set. The network’s behavior under various noise inputs is summarized in Table 1.

For a comprehensive evaluation, we selected an encoder with a noise level of 0.25 and a decoder with a noise level of 1.0 for participation in the following experiments.

### 4.4. Result Analysis

After completing the network training, we integrated it into the simulation model for verification. To verify the performance of the shape recognition algorithm and assess the quality of the network training, we separately evaluated the encoder and decoder networks.

**Figure 5:**
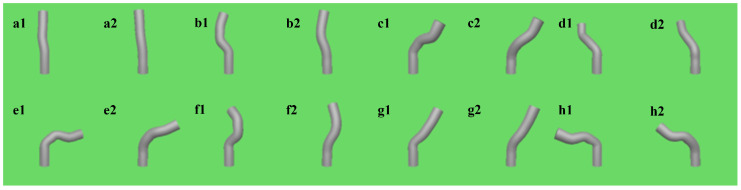
Shape Prediction Results of 8 Bending States: The left side (**a1**–**h1**) displays the actual shape, while the right side (**a2**–**h2**) presents the shape predicted by the network.

First, we validated the encoder network by confirming that it can directly compute the corresponding shape output from motor inputs, effectively achieving shape estimation analogous to solving forward kinematics. By manipulating the control handle, we influenced the relative positions of the joints based on the functional relationships defined in the simulation model, inducing deformations in the flexible continuum and obtaining the actual shape output. Simultaneously, the encoder network processed the input signal to calculate the joints’ relative positions, which were then mapped onto the bone joints to generate the simulation output. As illustrated in Figure 5, the left side represents the shape output from the simulation model, while the right side shows the shape predicted by the encoder network. Overall, the predicted shape closely approximates the actual shape, though certain errors are observed in the bending of certain joints. Notably, the prediction error increases when the instrument shape features a high degree of curvature.

**Figure 6:**
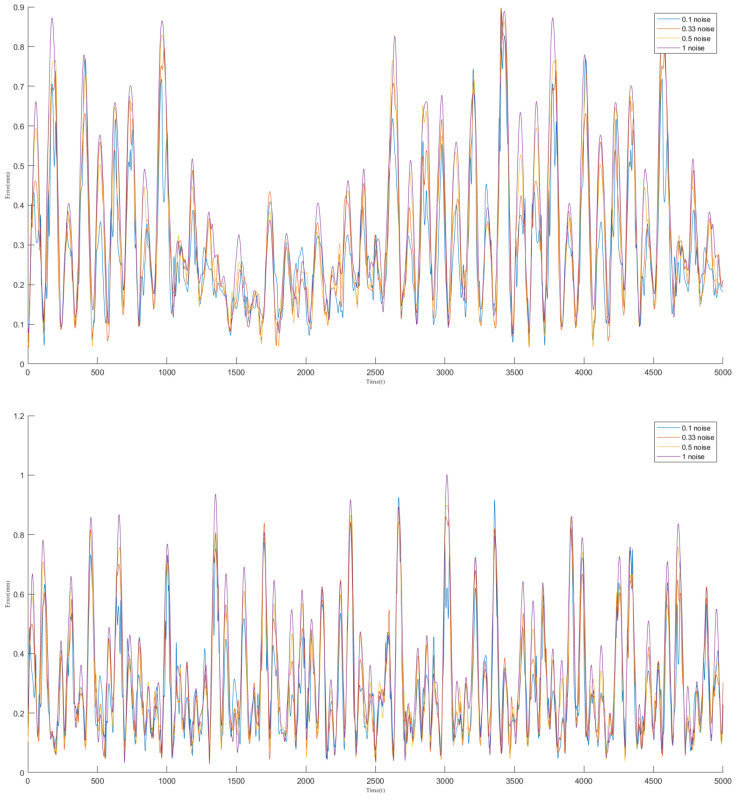
The average joint deviation for Validation Dataset 1 and Validation Dataset 2. The horizontal axis represents the sampling time points, while the vertical axis indicates the corresponding average joint deviation. The lines in different colors represent the network results under various noise training conditions.

To rigorously assess the prediction accuracy, we calculated the average joint deviation between the predicted results and the actual results in the validation dataset. As illustrated in Figure 6, the horizontal axis represents the sampling time, while the vertical axis shows the average joint deviation at each time step. The results demonstrate a strong correlation between the predicted outcome and the input signal, although the model’s performance degrades when predicting highly bent shapes.

**Figure 7:**
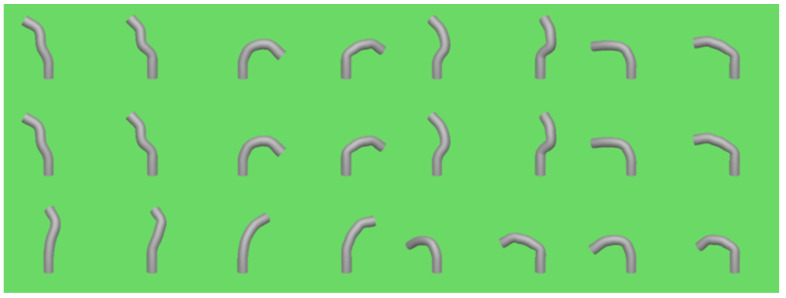
Shape control results with the actual shape displayed on the left and the reconstructed shape control results shown on the right.

**Figure 8:**
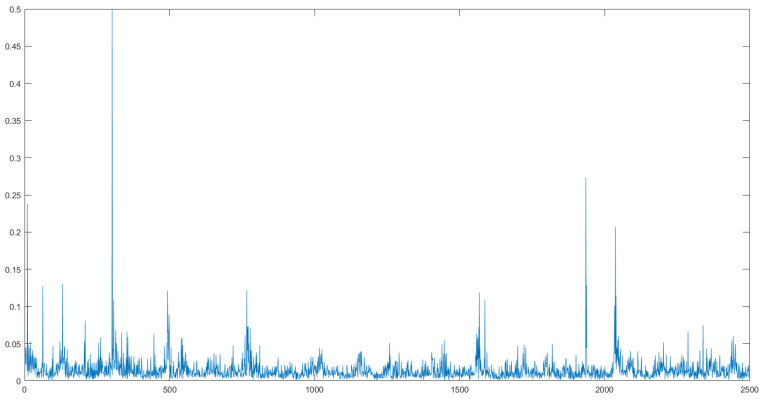
The shape control joint deviation curve.

Next, we validate the decoder network to confirm its ability to calculate appropriate motor inputs from shape information, effectively addressing an inverse kinematics problem. Given a specific target shape, the decoder generates corresponding handle input data, which is applied to a mathematical model to simulate the instrument’s shape. The simulated shape is then compared to the target shape to evaluate accuracy. The results are illustrated in Figure 7, where the left panel displays the target shape and the right panel shows the shape produced using motor inputs generated by the decoder and controlled via the simulation model. To validate the effectiveness of shape control, we calculate the mean joint position error over a certain sampling time, and the results are shown in Figure 8.

**Figure 9:**
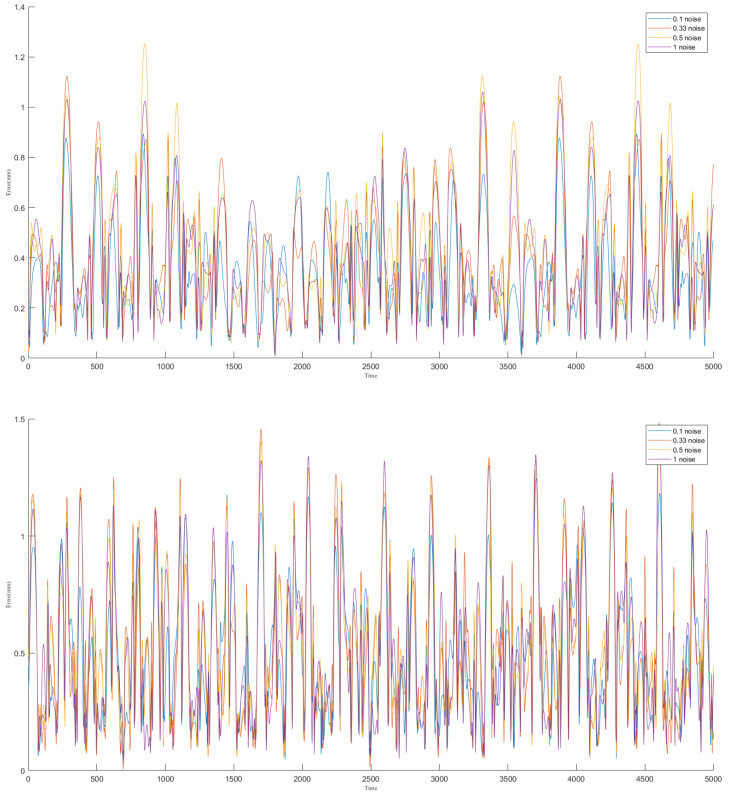
The average joint deviation for Validation Dataset 1 and Validation Dataset 2. The horizontal axis represents the sampling time points, while the vertical axis indicates the corresponding average joint deviation. The lines in different colors represent the network results under various noise training conditions.

During testing, we observed that the shape control algorithm performs more effectively under non-extreme bending conditions. To quantify this, we calculated the average joint position error over a specified sampling period, as shown in Figure 9. The horizontal axis represents the sampling time, and the vertical axis indicates the average joint deviation between the two shapes at each time point.

From Figure 7 and Figure 9, we infer that the decoder network efficiently translates shape data into appropriate motor inputs for devices with geometries similar to those in the dataset, enabling accurate shape control.

**Figure 10:**
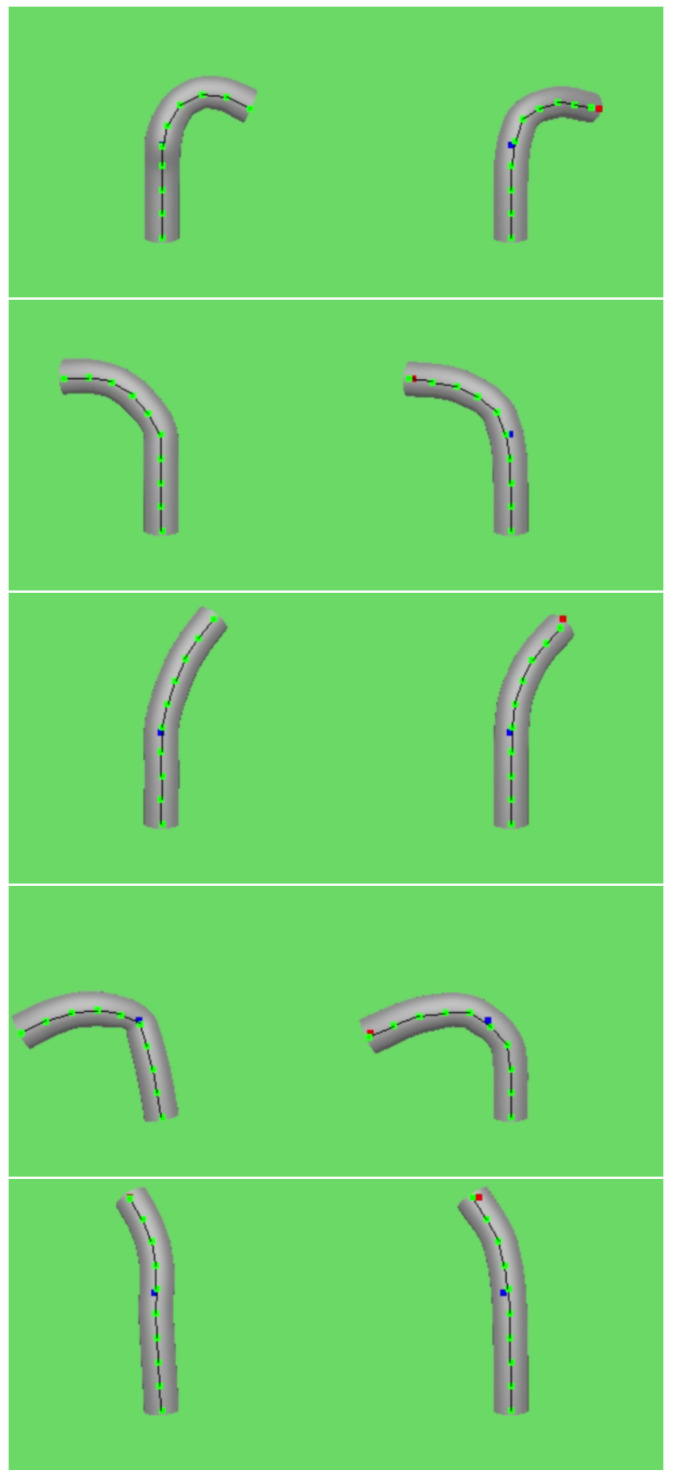
The results from the method in [31] are compared with our reconstruction. The red square represents the expected position of the object’s end joint, while the blue square represents the expected position of the object’s middle joint. On the left, we show the outcome using the method from [31] to control the joints of the flexible object, and on the right, we present the result after the network resolves the left posture.

To further evaluate the effectiveness of this shape control, we conducted two sets of experiments. In the first set, the method in [31] was used to directly manipulate the joints of flexible objects, guiding them to the desired positions in a simulation environment. The shape and orientation of these objects were then fed into the network, which generated motor inputs to control the flexible object. We compared the joint positions obtained using the method in [31] with those achieved by the network, as shown in Figure 10.

In the second set of experiments, we focused on reconstructing flexible objects in a real-world environment. Utilizing a binocular camera, we captured the shape of the flexible object and reconstructed the 3D coordinates of key points based on the camera’s position and orientation. These coordinates were then used to compute the object’s shape, which was input into the decoder network to generate the corresponding analog handle inputs. The reconstruction was finalized through a simulation-based mathematical model.

**Figure 11:**
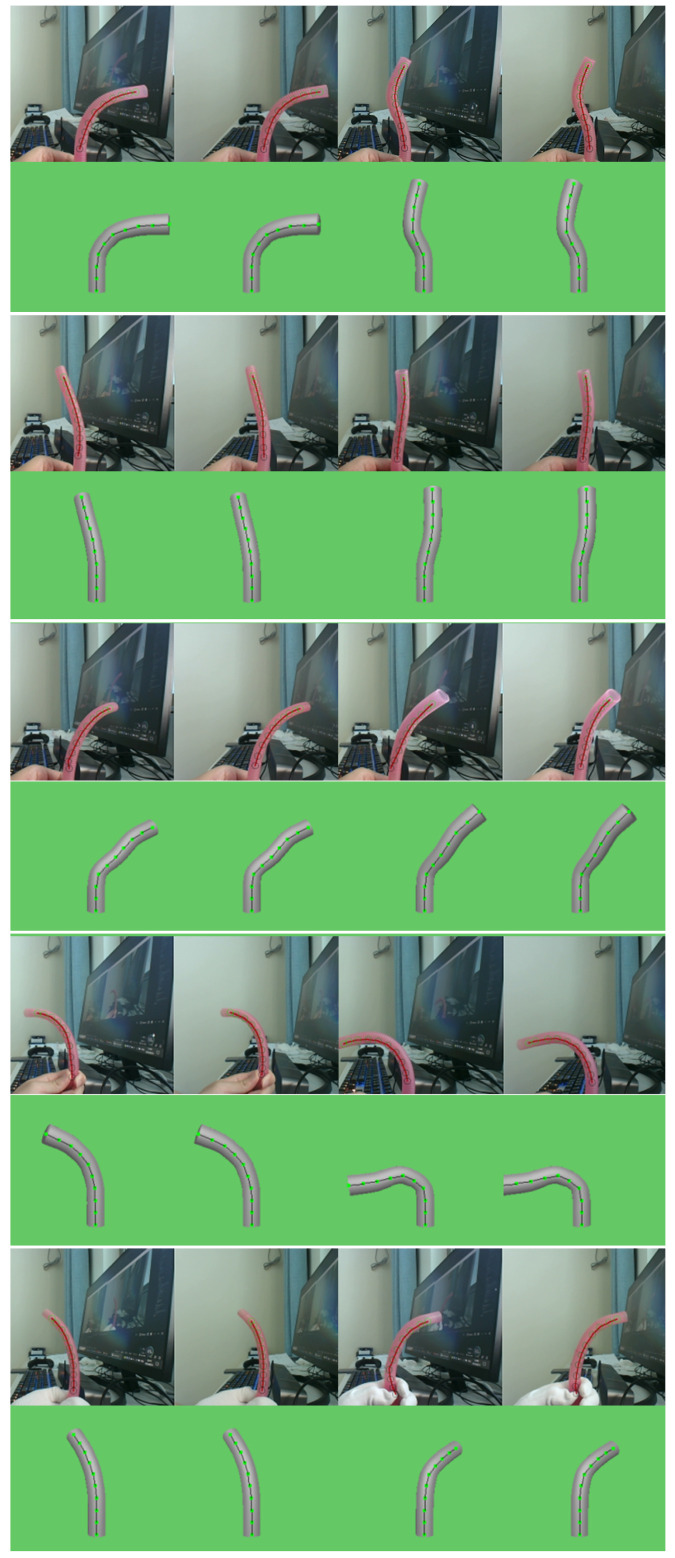
Real-world Shape Control Results: The shape of the flexible device in the real-world environment is replicated in a simulated environment, with its form controlled by a network-driven system.

As shown in Figure 11, the shape recognition and control algorithm successfully met its initial objectives. For a given instrument shape, we decoded the necessary motor inputs through the decoder network. By using these inputs, we can control the flexible surgical instrument to achieve the desired shape. In this manner, the decoder network enabled precise shape control of the flexible continuum.

## 5. Conclusions

This study introduces a neural network designed to tackle the challenge of end shape control for flexible surgical instruments, focusing on two key aspects: perception and control. The training data are derived from a specially developed simulation environment, with significant features identified through a shape recognition algorithm. To assess its effectiveness, the neural network is tested in practical applications, showcasing the potential of learning-based approaches for managing the shape of flexible surgical tools.

Our shape recognition algorithm performs effectively in cases involving slight to moderate instrument bending and is also capable of addressing more severe bending situations. By utilizing a trained encoder network, motor inputs are translated into shape outputs, ensuring precise shape estimation within the range of the training dataset. In addition, the network’s predictions provide valuable insights for input data beyond the training set. The decoder network, in turn, derives the motor inputs from the shape output, enabling precise control over the instrument’s shape. When the instrument’s shape deviates only slightly from the training data, the network successfully controls the device to achieve the desired shape.

In extreme bending scenarios, shape recognition becomes particularly challenging due to the significant deformations objects undergo. Utilizing topological invariants and curvature analysis may help preserve recognition accuracy, though this approach lies beyond the scope of the current paper and represents an avenue for future research. In addition, integrating data from multiple sensors—such as visual, haptic, or 3D scanning technologies—can improve shape recognition and will be a key focus of our future work.

## Figures and Tables

**Table 1:** MSE and actual variance of neural networks across various noise levels during training.

Noise	Network Name	MSE	Variance
0.1	encoder	0.28037	0.28059
0.33	encoder	0.30727	0.29850
0.5	encoder	0.32699	0.30925
1.0	encoder	0.35881	0.34083
0.1	decoder	0.35031	0.49787
0.33	decoder	0.41572	0.52816
0.5	decoder	0.45248	0.52723
1.0	decoder	0.42029	0.52670

## Data Availability

Data are contained within the article.
